# Cabergoline, Dopamine D2 Receptor Agonist, Prevents Neuronal Cell Death under Oxidative Stress via Reducing Excitotoxicity

**DOI:** 10.1371/journal.pone.0099271

**Published:** 2014-06-10

**Authors:** Haruki Odaka, Tadahiro Numakawa, Naoki Adachi, Yoshiko Ooshima, Shingo Nakajima, Yusuke Katanuma, Takafumi Inoue, Hiroshi Kunugi

**Affiliations:** 1 Department of Mental Disorder Research, National Institute of Neuroscience, National Center of Neurology and Psychiatry, Tokyo, Japan; 2 Department of Life Science and Medical Bioscience, School of Advanced Science and Engineering, Waseda University, Tokyo, Japan; 3 Core Research for Evolution Science and Technology Program (CREST), Japan Science and Technology Agency (JST), Tokyo, Japan; Universidad de Castilla-La Mancha, Spain

## Abstract

Several lines of evidence demonstrate that oxidative stress is involved in the pathogenesis of neurodegenerative diseases, including Parkinson's disease. Potent antioxidants may therefore be effective in the treatment of such diseases. Cabergoline, a dopamine D2 receptor agonist and antiparkinson drug, has been studied using several cell types including mesencephalic neurons, and is recognized as a potent radical scavenger. Here, we examined whether cabergoline exerts neuroprotective effects against oxidative stress through a receptor-mediated mechanism in cultured cortical neurons. We found that neuronal death induced by H_2_O_2_ exposure was inhibited by pretreatment with cabergoline, while this protective effect was eliminated in the presence of a dopamine D_2_ receptor inhibitor, spiperone. Activation of ERK1/2 by H_2_O_2_ was suppressed by cabergoline, and an ERK signaling pathway inhibitor, U0126, similarly protected cortical neurons from cell death. This suggested the ERK signaling pathway has a critical role in cabergoline-mediated neuroprotection. Furthermore, increased extracellular levels of glutamate induced by H_2_O_2_, which might contribute to ERK activation, were reduced by cabergoline, while inhibitors for NMDA receptor or L-type Ca^2+^ channel demonstrated a survival effect against H_2_O_2_. Interestingly, we found that cabergoline increased expression levels of glutamate transporters such as EAAC1. Taken together, these results suggest that cabergoline has a protective effect on cortical neurons via a receptor-mediated mechanism including repression of ERK1/2 activation and extracellular glutamate accumulation induced by H_2_O_2_.

## Introduction

Cabergoline is an ergot derived-dopamine D_2_-like receptor agonist that has high affinity for D_2_, D_3_, and 5-HT_2B_ receptors (K_i_ = 0.7, 1.5, and 1.2, respectively) [1]. Its property of having high affinity for D_2_ receptor is beneficial for dopamine replacement therapy of Parkinson disease (PD) [2], hyperprolactinemia [3], ovarian hyperstimulation syndrome [4], Cushing's disease [5], and restless legs syndrome [6]. Because cabergoline has a longer elimination half-life (63 to 109 h) compared with other D_2_-like receptor agonists, both a long-lasting clinical effect following single-dose administration [2], [7] and an improvement in the quality of life of patients with chronic diseases are expected.

Many studies have suggested that oxidative stress is involved in brain diseases such as ischemia [8], Alzheimer's disease (AD) [9], Huntington's disease (HD) [10], amyotrophic lateral sclerosis (ALS) [11], and PD [12]. Interestingly, neuroprotective effects of dopamine D_2_-like receptor agonists (including cabergoline) against oxidative stress have been reported [13]. An *in vivo* study of neuronal damage induced by intracerebroventricular (icv) injection of 6-OHDA, a neurotoxic compound that selectively damages dopaminergic neurons in male ICR mice, demonstrates that intraperitoneal (ip) administration of cabergoline for 7 days prevented nigrostriatal region dopaminergic neurons from cell death [14]. Cabergoline also protected SH-SY5Y neuroblastoma from cell death by oxygen-glucose deprivation even when cabergoline was administered after the induction of cell death [15]. In addition, the toxic effect of paraquat, which causes production of reactive oxidative species (ROS), on SH-SY5Y cells was reduced by co-incubation with cabergoline [16]. Other D_2_ receptor agonists, bromocriptine and quinpirole, also have marked neuroprotective effects against oxidative stress caused by glutamate, superoxide anions, and Ca^2+^ overload, in cultured mesencephalic neurons, although the protective effect depended on the duration of preincubation with these agonists prior to such toxic stimulants [17]. Importantly, several reports demonstrated that inhibition of the cabergoline effect using a D_2_-receptor antagonist was partial or not achieved [14], [16], suggesting cabergoline might mediate its protective effect through D_2_ receptor-dependent and -independent pathways. Previous studies demonstrated that cabergoline functions as a radical scavenger, and a direct antioxidant effect is recognized as the main action of cabergoline [14], [15], [18]. Although the possible contribution of receptor-mediated mechanisms such as upregulation of glutathione, an endogenous radical scavenger, have been shown [14], [16], the molecular mechanisms underlying D_2_ receptor-mediated neuroprotection by cabergoline are poorly understood.

Although the positive influence of D_2_ receptor agonists on mesencephalic neurons is well studied [13], [17], [19], neuronal responses in other brain regions is largely unknown. Besides the mesencephalon, the D_2_ receptor is expressed in several brain regions, including the hippocampus, olfactory forebrain, amygdale, and cerebral cortex [20]. Therefore, cabergoline could also affect these brain regions. Indeed, we previously reported that cabergoline increases hippocampal brain-derived neurotrophic factor (BDNF, an important regulator in the synaptic plasticity) and exerts an antidepressant effect in rats [21], [22], suggesting a beneficial effect of cabergoline on neuronal populations other than those in the mesencephalon. In the present study, we investigated the neuroprotective effect of cabergoline against oxidative stress caused by H_2_O_2_ in cultured cortical neurons, and found that significant neuroprotection occurred by a D_2_ receptor-mediated mechanism.

## Materials and Methods

### Primary cortical cultures

Primary cortical neurons were prepared as previously reported [23]. Postnatal 1–2 day-old Wistar rats were sacrificed by an overdose of isoflurane (Mylan, Tokyo, Japan) or diethyl ether (Wako, Osaka, Japan) inhalation and brains were quickly removed. Cerebral cortex tissues were dissected and treated with papain solution (PBS containing 9 units/ml papain and 200 units/ml DNase I) for 20 min at 37°C followed by dissociation with pipetting. After cell debris was removed by sterilizing filter (BD Falcon, CA, USA), cortical cells were diluted with culture medium (1∶1 mixture of Dulbecco's Modified Eagle's Medium and Ham's F-12 containing 5% fetal bovine serum (FBS), 5% horse serum (HS), 18 units/ml penicillin, and 18 mg/ml streptomycin). The dissociated cortical cells were plated at a cell density of 5×10^5^/cm^2^ on 3.5 cm dishes (for western blotting), 24-well plates (for quantitative reverse transcription polymerase chain reaction (qRT-PCR) and glutamate measurement), 48-well plates (for MTT [3-(4,5- Dimetylthial-2-yl)-2,5- Diphenyltetrazolium Bromide] assay, and calcein-assay), and glass-bottom dishes (for immunostaining). All dishes or plates were coated with polyethylenimine. To exclude an influence of glial cells in this study, AraC (Cytarabine, 2 µM), a potent inhibitor of glial proliferation, was added at day 1 of the *in vitro* culture (DIV1). This study was approved by the Animal Ethics Committee of the National Institute of Neuroscience, National Center of Neurology and Psychiatry, Japan. All animal experiments in this study were carried out in accordance with the guidelines of the committee.

### Drug treatment

Cabergoline (10 µM; except for experiments of dose-dependency, Tocris Bioscience, Bristol, UK) was applied to cortical cells at DIV 6-7. After 24-hour cabergoline treatment (except for examination of pretreatment time-dependency of cabergoline), H_2_O_2_ (50 µM; except for the dose-dependency of H_2_O_2_, Wako) was added. All inhibitors and antagonists, including spiperone (Abcam, Bristol, UK), U0126 (Promega, WI, USA), SB203580 (Millipore, MA, USA), SP600125 (Wako), AP5 (Tocris bioscience), and nifedipine (Sigma-Aldrich, MO, USA) were applied 20 min before cabergoline or H_2_O_2_ addition. L-glutamate (Wako) was added at DIV 7–8 for cell death induction.

### MTT assay

Cell survival rate was measured by MTT assay as previously reported [23]. After the indicated treatment with drugs was completed, culture medium was replaced with 200 µl fresh medium containing 40 µl MTT solution (2.5 mg/ml, diluted in PBS) and cells were incubated at 37°C for 1.5–2.5 hours. Then, 200 µl lysis buffer containing isopropyl alcohol was applied to each well and mixed by pipetting. Each sample was moved to a 96-well plate and its absorbance at 570 nm was measured using an iMark Micro plate leader (Bio-Rad Laboratories Inc., CA, USA). Cell survival rate was quantitated by absorbance measurement, because MTT (yellow) is deoxidized to formazan (violet) in proportion to mitochondrial activity.

### Calcein-assay

To assess cell viability by another method, the calcein-assay was also performed using a Cell Counting Kit-F (Dojindo, Kumamoto, Japan) according to the manufacturer's protocol with some modifications. Briefly, after cultured cells were washed with PBS, they were exposed to 50-fold diluted Cell Counting Kit-F Solution (Calcein-AM solution) for 20 min at room temperature. After washing several times and replacement with PBS, the fluorescent intensity (485/535 nm) was measured using a 2030 ARVO X-2 Multilabel Reader (PerkinElmer Japan Co., Ltd., Kanagawa, Japan). Cell viability can be quantitated, because calcein-AM (membrane-permeable, nonfluorescent) is hydrolyzed to calcein (membrane-impermeable, fluorescent) by esterase activity in surviving cells.

### Immunostaining

Neuronal survival was detected by immunostaining with anti-MAP2 (Microtubule-associated protein 2) antibody. After cultured cells were washed with cold PBS, cells were fixed with 4% paraformaldehyde at 24°C for 20 min. After blocking with PBS containing 10% FBS and 0.5% Triton-X for 30 min, cells were exposed to PBS containing 10% FBS and anti-MAP2 antibody (1∶500, Sigma-Aldrich) at 4°C overnight. As a secondary antibody, Alexa Fluor 488 mouse IgG_1_ (1/200, Life Technologies, CA, USA) was added at 24°C for 1 hour. Immunoreactivity was assessed by Axio Observer.Z1 fluorescence microscopy (ZEISS, Germany). To detect the apoptotic phenotype of cells, we performed nuclear staining using Hoechst 33342 (Molecular Probes, OR, USA). After fixation of cultured cells with 4% paraformaldehyde, cells were incubated with 5 µg/ml Hoechst 33342 at room temperature for 15 min. Then, cells were washed with PBS and condensed nuclei were detected.

### Western Blotting

Cortical cells were washed with cold PBS and stored at −20°C until assay use. The cells were lysed with lysis buffer (1% Sodium dodecyl sulfate, 20 mM Tris-HCl (pH 7.4), 5 mM EDTA (pH 8.0), 10 mM NaF, 2 mM Na_3_VO_4_, and 1 mM phenylmethylsulfonyl fluoride). The collected lysed samples were heated at 100°C for 3 min, then sonicated and centrifuged (15,000 rpm, 30 min, 24°C). The supernatant was used for determining total protein concentration using a BCA Protein Assay Kit (Thermo Scientific, MA, USA). The same amount of protein from each sample was separated on an 8 or 10% polyacrylamide gel by electrophoresis, and transferred onto polyvinylidene difluoride (PVDF) membranes using Semi-Dry Transfer Cell (BioRad). The membrane was blocked in 5% non-fat dried milk solution for 1 hour, and incubated with primary antibody at 4°C, overnight. Subsequently, the membrane was washed with Tris-Buffered Saline (TBS) for 1 hour and incubated with secondary antibody at room temperature for 1 hour. After washing with TBS for 1 hour, immunoreactivity was visualized by ImmunoStar Regents (Wako). To obtain images of immunoreactivity, exposure to X-ray film (GE Healthcare, WI, USA) or acquisition using EZ Capture II/ST (ATTO, Tokyo, Japan) was performed. The density of each band was quantified using Lane & Spot Analyzer software (ATTO). The following antibodies were used as primary antibodies: anti-dopamine D2 receptor (1∶500, Millipore), anti-synapsinI (1∶1000, Millipore), anti-pERK (1∶1000, Cell Signaling, MA, USA), anti-ERK (1∶1000, Cell Signaling), anti-pJNK (1∶1000, Cell Signaling), anti-JNK (1∶1000, Cell Signaling), anti-pp38 (1∶500, Cell Signaling), anti-p38 (1∶1000, Cell Signaling), anti-NR2A (for total 1∶500, for cell surface 1∶200, Sigma-Aldrich), anti-NR2B (for total 1∶500, for cell surface 1∶200, Sigma-Aldrich), anti-GluR1 (1∶500, Millipore), anti-GluR2/3 (1∶500, Millipore), anti-EAAC1 (1∶1000, Alpha Diagnostic International, San Antonio, USA), anti-GLT-1 (1∶500, Santa Cruz Biotechnology, Santa Cruz, USA), and anti-βactin (1∶5000, Sigma-Aldrich) antibodies. Rabbit IgG (H&L) Secondary Antibody Peroxidase Conjugated Properties (Rockland), or Peroxidase-AffiniPure Goat Anti-Mouse IgG (Jackson Immunoresearch Labs Inc.,) were used as secondary antibodies.

### Cell surface labeling

Cell surface protein levels were measured using surface labeling and immunoprecipitation [23]. Cortical cells were washed with cold PBS 3 times on ice and exposed to Sulfo-NHS-LC-Biotin (Thermo Scientific) at 4°C for 30 min. Afterwards, cells were washed with cold PBS containing glycine (100 mM) 3 times and stored at −20°C before performing lysis. The cells were lysed with RIPA lysis buffer (1% Triton X100, 20 mM Tris-HCl (pH 7.4), 5 mM EDTA (pH 8.0), 10 mM NaF, 2 mM Na_3_VO_4_, and 1 mM phenylmethylsulfonyl fluoride) on ice. The homogenate was rotated at 4°C for 3 hours before centrifugation (15,000 rpm, 30 min, 4°C). After the supernatant was collected and its protein concentration was measured, equal amounts of protein were mixed with UltraLink Immobilized NeutrAvidin Protein Plus, conjugated with agarose beads (Thermo Scientific), and rotated at 4°C overnight. The supernatant was removed by centrifugation (5000 rpm, 5 min, 4°C), and bead pellets were washed with lysis buffer. Finally, proteins attached to beads were eluted by heating at 100°C for 5 min and each sample was used for SDS-PAGE.

### Glutamate release measurement

Measurement of glutamate content in the collection buffer by high performance liquid chromatography (HPLC, Shimazu Co., Japan) was performed as previously reported [23]. Briefly, culture medium was removed and each well of a culture plate was washed with KRH buffer (130 mM NaCl, 5 mM KCl, 1.2 mM NaH_2_PO_4_, 1.8 mM CaCl_2_, 10 mM glucose, 1% BSA, and 25 mM HEPES, pH 7.4) 3 times. Then, KRH buffer was applied for 20 min and collected as the baseline release of glutamate. Additionally, KRH buffer containing 50 µM H_2_O_2_ was added for 20 min for H_2_O_2_-induced release.

### RNA extracts, Reverse Transcription PCR, and Real Time PCR

Extraction of RNA was performed using *mir*vana miRNA Isolation Kit (Life Technologies) according to the manufacturer's protocol. Reverse Transcription PCR was performed using equal amounts of total RNA with SuperScript VILO cDNA Synthesis Kit (Life Technologies) (25°C for 10 min, 42°C for 60 min, 85°C for 5 min, and stored at 4°C). Then, synthesized cDNA was amplified using specific primers from TaqMan Gene Expression Assays (Life Technologies). Real-time PCR was performed by incubation of PCR reaction mixtures at 50°C for 2 min, 95°C for 10 min, followed by 40 PCR cycles (95°C for 15 sec, 60°C for 1 min) in an ABI Prism 7000 (Applied Biosystems). We purchased specific primers for BDNF (Rn02531967_s1) and Glyceraldehyde-3-phosphate dehydrogenase (GAPDH (4352338E)), from Applied Biosystems. Levels of BDNF mRNA were normalized to levels of GAPDH mRNA.

### Statistics

All data are expressed as means ± SD. Statistical significance was estimated for single analysis by Bonferroni test or for multiple analyses by ANOVA (SPSS Japan, Tokyo, Japan). Two tailed *P-*values less than 0.05 were regarded as statistically significant.

## Results

### Neuroprotective effects of cabergoline on cultured cortical neurons

**Figure 1:**
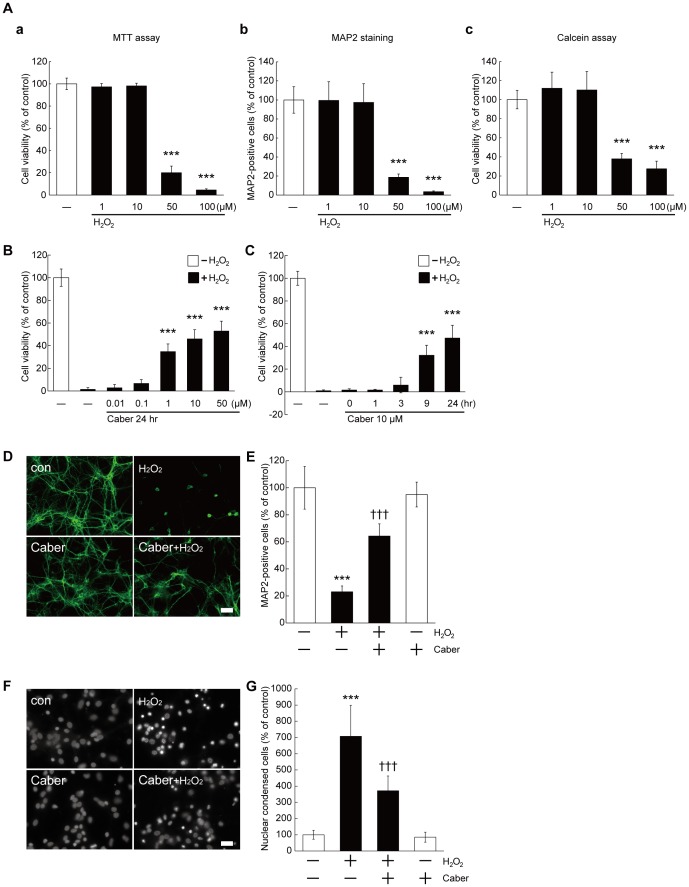
Cabergoline prevented H_2_O_2_-induced cell death in a dose- and pretreatment-time-dependent manner. (A) H_2_O_2_ application dose-dependently caused neuronal cell death. H_2_O_2_ was applied to DIV 7–8 neurons at 1, 10, 50, or 100 µM (for 9–12 hours). Cell viability was measured by MTT assay (a), MAP2 staining (b) or Calcein assay (c). The data represent mean ± SD (a: n = 7–16, c: n = 7–14, n indicates the number of wells used per plate for each experimental condition; b: n = 24, n indicates the number of randomly selected fields from 8 coverslips for each experimental condition). ***P<0.001 vs. - H_2_O_2_ (one-way ANOVA). (B) Pretreatment of cabergoline inhibited H_2_O_2_-induced cell death in a dose-dependent manner. Cabergoline was applied at DIV 6–7 (0.01, 0.1, 1, 10, or 50 µM, for 24 hours). Then, 50 µM of H_2_O_2_ was added followed by MTT assay. The data represent mean ± SD (n = 5–12). ***P<0.001 vs. + H_2_O_2_ - Caber (two-way ANOVA). Caber: Cabergoline. (C) Neuroprotection by cabergoline depended on the duration of the treatment time. Pretreatment with cabergoline (10 µM) was performed at 0, 1, 3, 9 or 24 hours before addition of 50 µM H_2_O_2_ followed by MTT assay. The data represent mean ± SD (n = 4–12). ***P<0.001 vs. + H_2_O_2_ - Caber (two-way ANOVA). (D), (E) Immunostaining with MAP2 antibody was performed. Pretreatment with cabergoline (10 µM) was performed 24 hours before H_2_O_2_ addition. *Scale bar*, 50 µm. E, Quantification analysis by counting the number of MAP2-positive cells is shown. The data represent mean ± SD (n = 12, n indicates the number of randomly selected fields from 4 coverslips for each experimental condition). ***P<0.001 vs. - H_2_O_2_ - Caber, †††*P*<0.001 vs. + H_2_O_2_ - Caber (two-way ANOVA). (F), (G) Cabergoline suppressed H_2_O_2_-induced apoptotic nuclear condensation. Nuclear staining with Hoechst 33342 was performed. Pretreatment with cabergoline (10 µM) was performed 24 hours before H_2_O_2_ addition. *Scale bar*, 25 µm. G, Quantification analysis by counting the number of condensed nuclei is shown. The data represent mean ± SD (n = 15, n indicates the number of randomly selected fields from 5 coverslips for each experimental condition). ***P<0.001 vs. - H_2_O_2_ - Caber, †††*P*<0.001 vs. + H_2_O_2_ - Caber (two-way ANOVA).

To examine the potential protective effect of cabergoline on cultured cortical neurons under oxidative stress, we initially determined the dose-dependent induction of cell death by H_2_O_2_ (1–100 µM) using three distinct methods including MTT assay, MAP2 staining, and Calcein-AM assay. As shown in Fig. 1A, a significant decrease in cell viability caused by H_2_O_2_ at a concentration of 50 µM was detected by these three methods, although further cell death was induced at 100 µM. Because the dose-dependency of H_2_O_2_ for cell death estimated by the three methods correlated with each other, we used the MTT assay for all further experiments because of its higher availability. As 50 µM H_2_O_2_ induced marked cell death compared with lower doses, this concentration was used for the following experiments. To examine whether cabergoline exerted neuroprotection against H_2_O_2_, we applied cabergoline at various concentrations (0.01–50 µM) for 24 hours before H_2_O_2_ stimulation. Pretreatment with cabergoline inhibited H_2_O_2_-induced neuronal cell death in a dose-dependent manner (Fig. 1B). In the following experiments, we used 10 µM of cabergoline to investigate its neuroprotective effects. Cells were pre-incubated with cabergoline for different times (0–24 hours), with the maximum protection observed for 24 hour pretreatment (Fig. 1C). We confirmed that the application of cabergoline alone (without H_2_O_2_) had no effect on cell viability (Fig. S1). Therefore, cabergoline was added 24 hours prior to H_2_O_2_ application in this study. MAP2 staining revealed that cabergoline significantly suppressed the loss of neurons caused by H_2_O_2_ incubation (Fig. 1D, E). The detection of apoptotic nuclear condensation suggested that cabergoline prevented apoptotic cell death following H_2_O_2_ exposure (Fig. 1F, G).

### Expression of D_2_ receptor in cortical neurons and receptor dependency of neuroprotection by cabergoline

**Figure 2:**
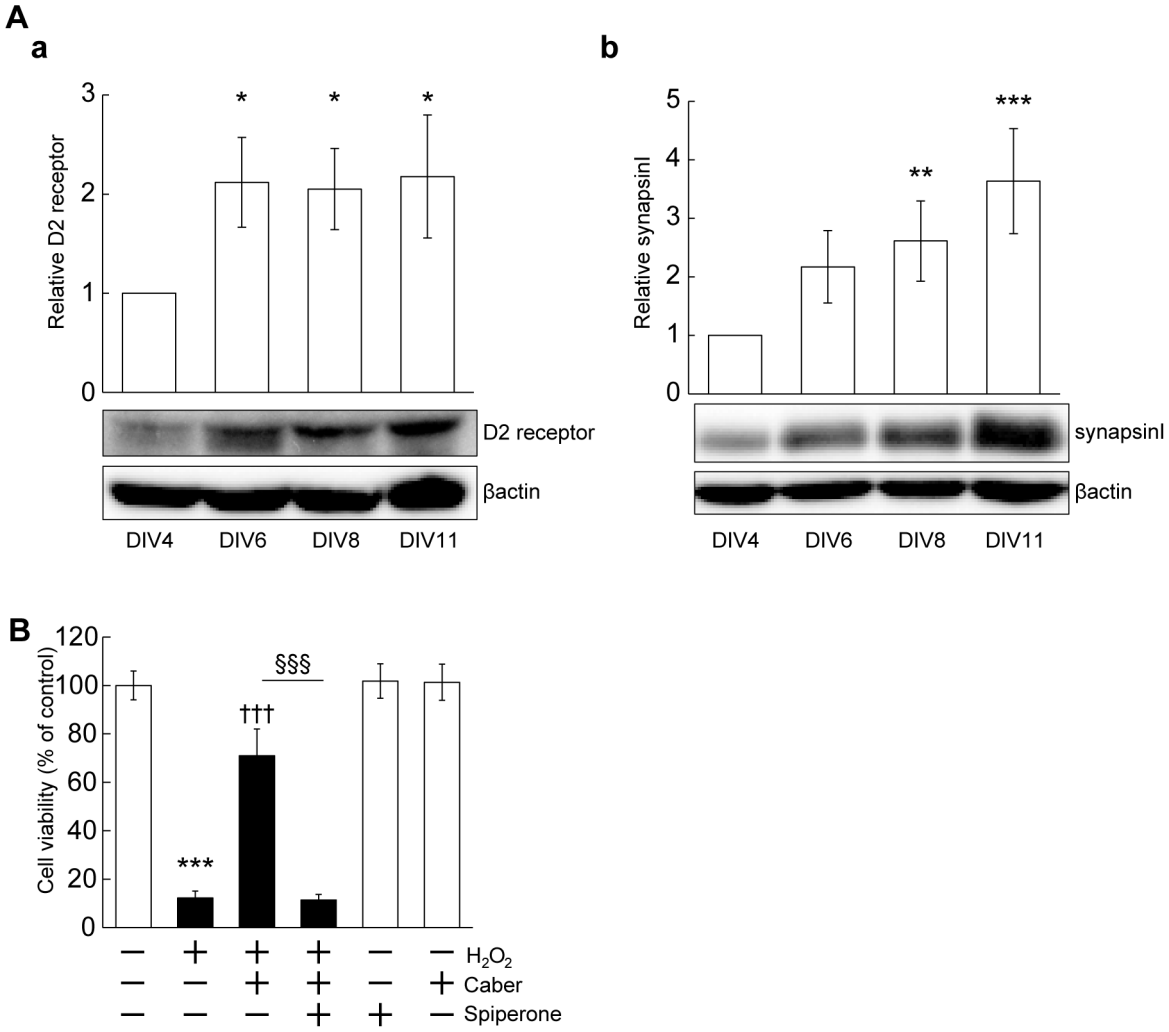
Cabergoline exerted neuroprotective effect via D_2_ receptor-mediated mechanism. (A) The expression of D_2_ receptor and of synapsinI during *in vitro* maturation (DIV 4–11). a: The change in protein levels of D_2_ receptor. *P<0.05 vs. DIV 4 (one-way ANOVA). b: The change of synapsinI expression during *in vitro* maturation. The data represent mean ± SD (a: n = 4, b: n = 5, n indicates the number of experiments using independent cultures). ***P<0.001, **P<0.01 vs. DIV 4 (one-way ANOVA). Relative values after normalizing to that of DIV4 are shown. (B) Spiperone inhibited neuroprotection by cabergoline. Spiperone (10 µM) was applied 20 min before cabergoline (10 µM) treatment, followed by MTT assay. The data represent mean ± SD (n = 6–12). ***P<0.001 vs. - H_2_O_2_ - Caber - spiperone, †††*P*<0.001 vs. + H_2_O_2_ - Caber - spiperone (three-way ANOVA).

Cabergoline acts as a potent agonist of D_2_, D_3_ and 5-HT_2B_ receptors [1]. D_2_ receptor is broadly expressed in the central nervous system (CNS) including the cerebral cortex, while D_3_ and 5-HT_2B_ receptors are relatively restricted and poorly expressed in the cortical region *in vivo* [20], [24], [25]. We examined the expression of D_2_ receptor during *in vitro* maturation, and confirmed its higher expression at DIV 6–11 compared with that at DIV4 (Fig. 2Aa). As a marker of synaptic maturation in our cortical cultures, an increase in levels of synapsinI was also detected (Fig. 2Ab).

In mesencephalic neurons and a neural cell line, it was reported that receptor dependency of neuroprotection by cabergoline was limited or was not confirmed [14], [15], [16], [18]. Therefore, the effect of spiperone, a potent D_2_-like and 5-HT receptor antagonist, on cabergoline-dependent neuroprotection was examined. Interestingly, spiperone abolished the neuroprotective effect of cabergoline (Fig. 2B). The addition of spiperone alone had no toxic influence in the presence or absence of H_2_O_2_ (Fig. 2B, Fig. S2). These results suggest that a receptor-mediated mechanism is involved in the survival-promoting effect of cabergoline.

### Involvement of ERK and p38, but not JNK, signaling in cell death caused by H_2_O_2_

**Figure 3:**
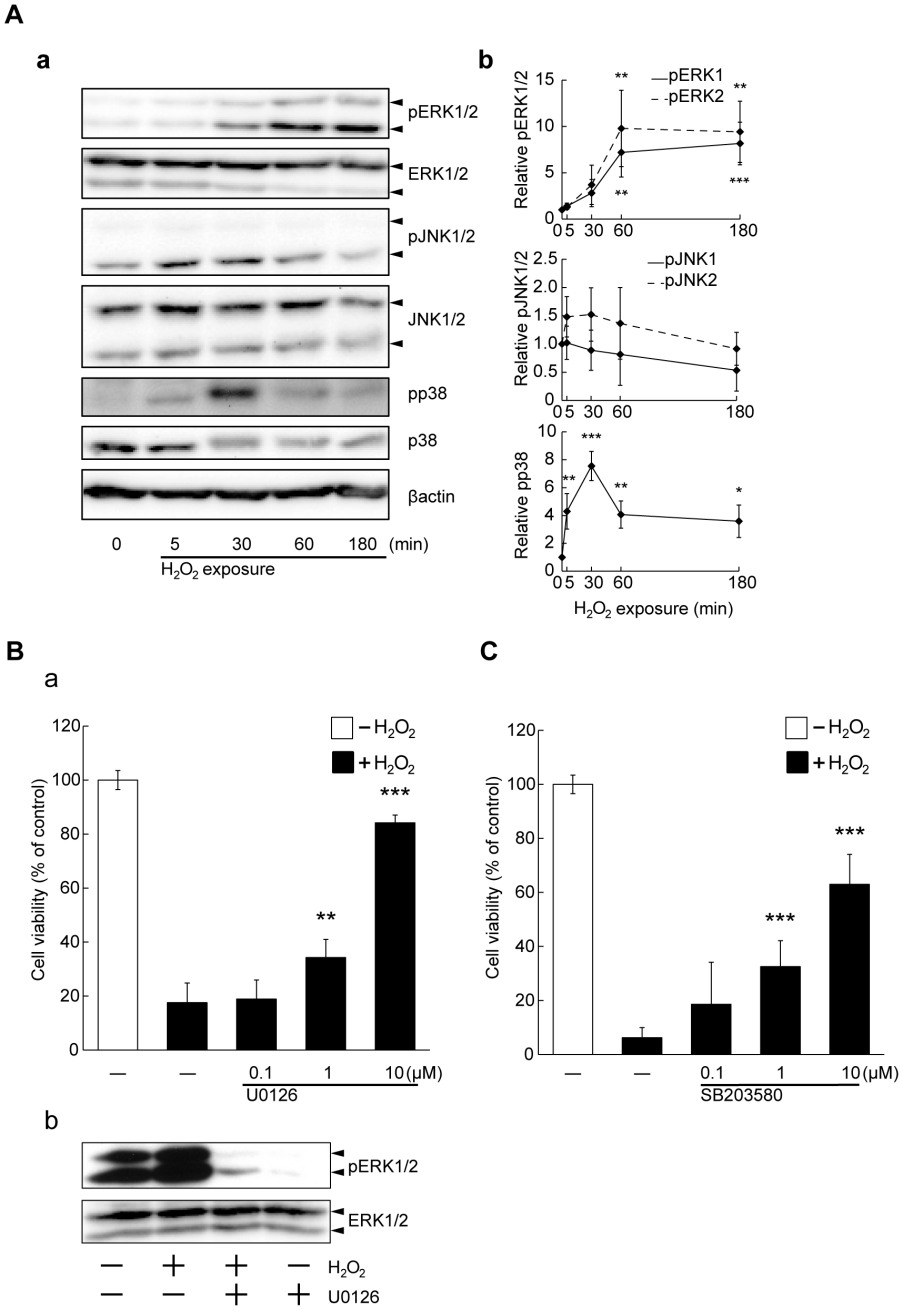
H_2_O_2_ stimulated activation of MAPK death signaling. (A) Time-course of H_2_O_2_-induced MAPK cascades. Cortical neurons were exposed to H_2_O_2_ (50 µM). a: 5, 30, 60 or 180 min later, phosphorylated ERK1/2, JNK1/2 and p38 were detected by western blotting. After that, the membranes were stripped and re-blotted with anti-total ERK1/2, JNK1/2, and p38 antibodies. b: Quantification of phosphorylated ERK1/2, JNK1/2 and p38 levels. All data represent mean ± SD (n = 4). ***P<0.001, **<0.01, *<0.05 vs. 0 min (one-way ANOVA). (B) a: U0126, an ERK signal inhibitor, suppressed H_2_O_2_-induced neuronal cell death in a dose-dependent manner. U0126 was added at 0.1, 1, or 10 µM. After 20 min, H_2_O_2_ application was performed, followed by MTT assay. The data represent mean ± SD (n = 4). ***P<0.001, **<0.01 vs. + H_2_O_2_ - U0126 (two-way ANOVA). b: U0126 suppressed phosphorylation of ERK1/2. U0126 was added at 10 µM. After 20 min, cortical neurons were exposed to H_2_O_2_ (50 µM) for 1 hour. After pERK1/2 was detected by western blotting, the membranes were stripped and re-blotted with anti-total ERK1/2 antibody. (C) SB203580, a p38 inhibitor, suppressed H_2_O_2_-induced neuronal cell death. SB203580 was added at 0.1, 1, or 10 µM followed by MTT assay. The data represent mean ± SD (n = 8). ***P<0.001 vs. + H_2_O_2_ - SB203580 (two-way ANOVA).

It is well known that mitogen-activated protein kinase (MAPK) cascades consist of three signaling pathways, including ERK, JNK, and p38, that contribute to cell proliferation, differentiation, survival, and death. Oxidative stress activates MAPK cascades in various cell populations, although their contribution to apoptosis depends on the specificity of cell population or stress stimuli [26]–[28]. We measured phosphorylated ERK1/2 (pERK1/2), pJNK1/2, and pp38 levels after H_2_O_2_ application. Increased levels of pERK1/2 were observed at 1 hour after H_2_O_2_ stimulation and persisted for 3 hours (Fig. 3A). Levels of pp38 reached a maximum level at 30 min after H_2_O_2_ addition, while pJNK was unchanged (Fig. 3A). Importantly, we found that U0126, an inhibitor of ERK signaling, decreased the induction of cell death and activation of ERK1/2 caused by H_2_O_2_ (Fig. 3B). SB203580, an inhibitor of p38 signaling, also markedly suppressed cell death induced by H_2_O_2_ (Fig. 3C). We confirmed no effect on cell viability with U0126 or SB203580 treatment in the absence of H_2_O_2_ (Fig. S3). In contrast, SP600125 (an inhibitor of JNK signaling) had a slight protective effect against H_2_O_2_ (Fig. S4). Thus, the ERK and p38 pathways might have important roles in cell death induced by H_2_O_2_.

### Cabergoline suppressed death signaling stimulated by H_2_O_2_

**Figure 4:**
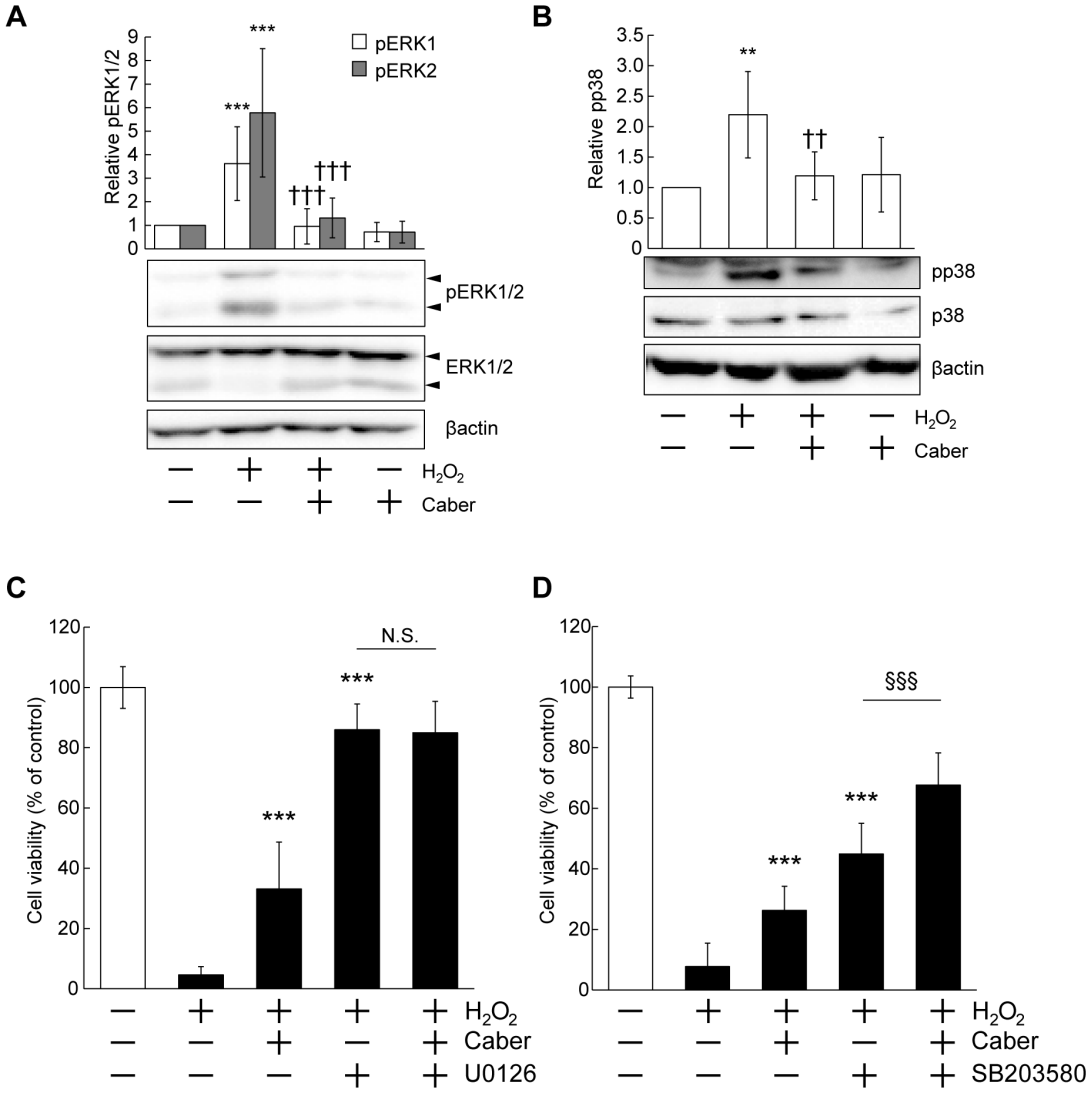
Cabergoline reduced death signaling by H_2_O_2_. (A) (B) Pretreatment of cabergoline inhibited H_2_O_2_-induced activation of ERK1/2 and p38. Cabergoline was added at 10 µM. After 24 hours, cortical neurons were exposed to H_2_O_2_ (50 µM) for 1 hour (pERK1/2) or 30 min (pp38), followed by western blotting. The membranes were stripped, and re-blotted with anti-total ERK1/2 and p38 antibodies. *Upper*: Quantification of phosphorylated ERK1/2 or p38 levels. All data represent mean ± SD (pERK1/2: n = 7, pp38: n = 5). ***P<0.001, **P<0.01 vs. - H_2_O_2_ - Caber, †††*P*<0.001, ††*P*<0.01 vs. + H_2_O_2_ - Caber (two-way ANOVA). (C) Co-treatment of cabergoline and U0126 had no additive effect on neuroprotection. U0126 (10 µM) was added 20 min before cabergoline (10 µM) treatment, followed by MTT assay. The data represent mean ± SD (n = 9–12). ***P<0.001 vs. + H_2_O_2_ - Caber - U0126 (three-way ANOVA). (D) Co-treatment of cabergoline and SB203580 showed a significant additive effect. SB203580 (10 µM) was added 20 min before cabergoline (10 µM) treatment. The data represent mean ± SD (n = 9–12). ***P<0.001 vs. + H_2_O_2_ - Caber - SB203580 (three-way ANOVA).

To reveal the molecular mechanisms underlying the neuroprotective effect of cabergoline, we assessed the possibility that cabergoline repressed H_2_O_2_-induced ERK and p38 phosphorylation. As expected, cabergoline significantly inhibited both ERK and p38 phosphorylation (Fig. 4A, B). We performed co-application of U0126 or SB203580 in the presence of cabergoline. No additional or synergistic influences were observed by the co-application of U0126 with cabergoline compared with the application of U0126 alone (Fig. 4C). However, co-treatment of cabergoline and SB203580 had an additional effect compared with treatment of cabergoline or SB203580 alone (Fig. 4D). Taken together, cabergoline might exert neuroprotective effects predominantly via the suppression of ERK signaling.

### Cabergoline reduced excitatory insults induced by H_2_O_2_

**Figure 5:**
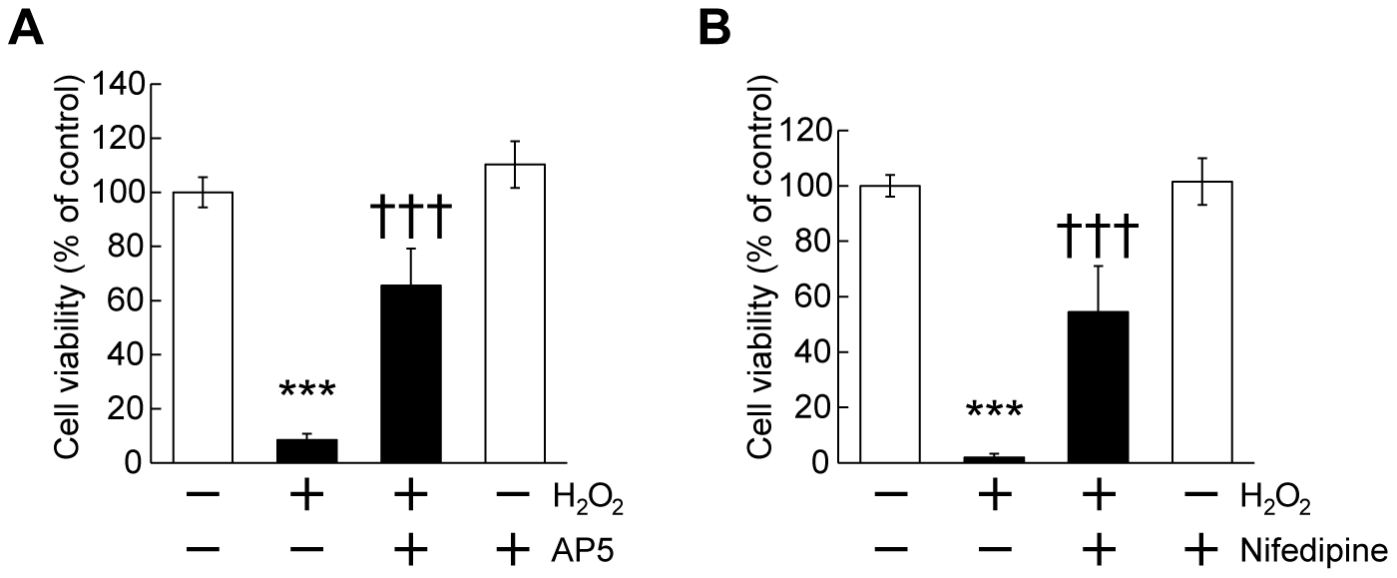
H_2_O_2_ exposure caused neuronal cell death via glutamate-mediated excitotoxicity. (A) AP5, an NMDA receptor blocker, suppressed H_2_O_2_-induced cell death. AP5 (10 µM) was added 20 min before H_2_O_2_ (50 µM) application, followed by MTT assay. The data represent mean ± SD (n = 6–12). ***P<0.001 vs. - H_2_O_2_ - AP5, †††*P*<0.001 vs. + H_2_O_2_ - AP5 (two-way ANOVA). (B) Nifedipine, a L-type Ca^2+^ channel blocker, inhibited cell death. Nifedipine (10 µM) was added 20 min before H_2_O_2_ (50 µM) application. The data represent mean ± SD (n = 6–12). ***P<0.001 vs. - H_2_O_2_ - Nifedipine, †††*P*<0.001 vs. + H_2_O_2_ - Nifedipine (two-way ANOVA).

To further clarify the mechanism underlying cabergoline functions, we focused on excitotoxicity mediated by the glutamate system under oxidative stress because H_2_O_2_ exposure of cortical neurons leads to an accumulation of extracellular glutamate and resultant cell death via activation of NMDA receptors (Ca^2+^-permeable glutamate receptor) [29], [30]. It was reported that glutamate-mediated excitotoxicity requires Ca^2+^ influx via NMDA receptors and/or voltage-dependent Ca^2+^ channels [31]. As shown in Fig.5, we observed that both AP5 (an NMDA receptor blocker) and nifedipine (an L-type Ca^2+^ channel blocker) reduced cell death induced by H_2_O_2_ (Fig. 5A, B). In our culture experiments, a dose-dependent toxic effect of glutamate was also confirmed (Fig. S5).

**Figure 6:**
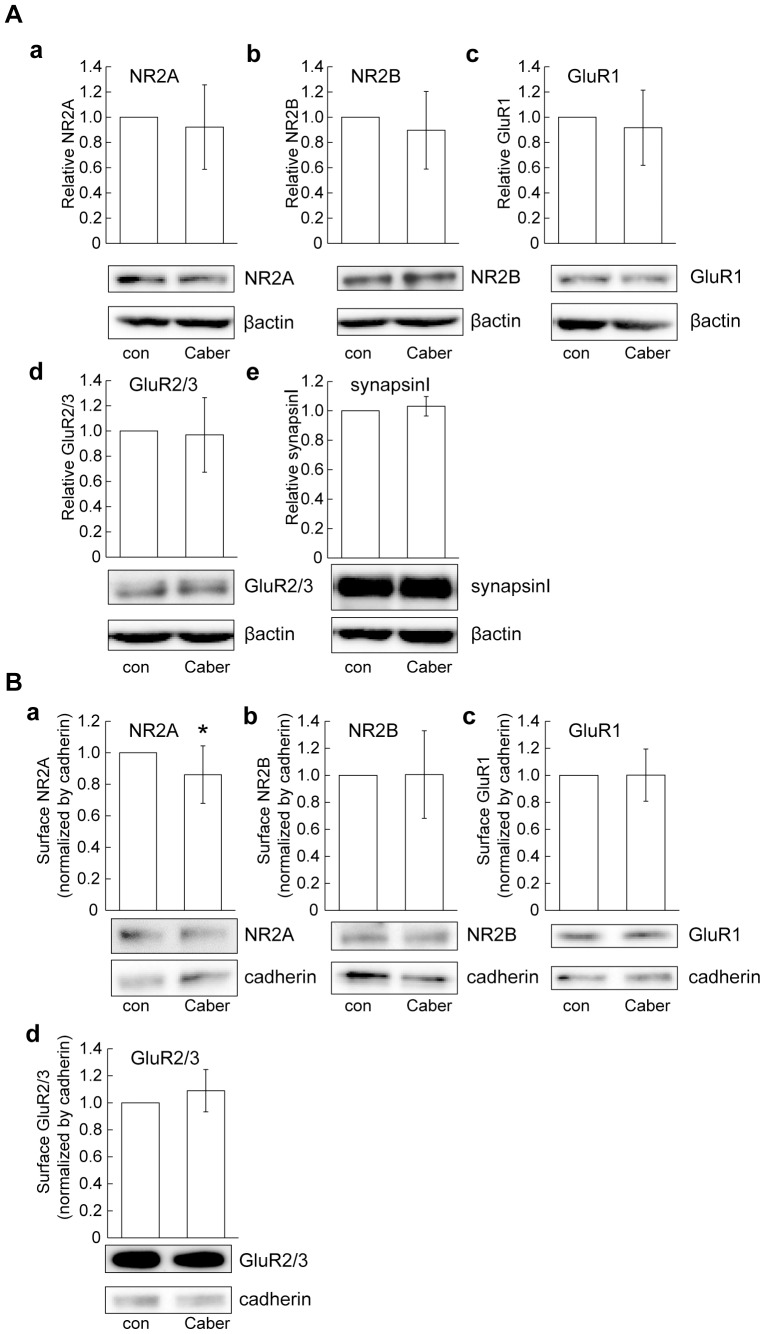
Levels of cell surface and total expression of glutamate receptors after cabergoline treatment. (A) No change in total expression levels of glutamate receptor subunits after cabergoline treatment. Total expression levels of NR2A, NR2B, GluR1, GluR2/3 and synapsinI after cabergoline treatment (10 µM for 24 hours). SynapsinI: pre-synaptic protein. *Upper*: Quantification of each protein was carried out. All data represent mean ± SD (n = 8). Statistics determined by *t*-test. (B) Cell surface expression levels of NR2A, NR2B, GluR1 and GluR2/3. *Uppe*r: Quantification of each protein was performed by normalizing to expression of cadherin (cell surface protein). All data represent mean ± SD (NR2A: n = 9, NR2B: n = 6, GluR1, GluR2/3: n = 8). *P<0.05 vs. - Caber (t-test).

As the contribution to excitotoxicity mediated by glutamate was revealed in our system, we measured the effect of cabergoline on expression levels of glutamate receptor subunits including NR2A, NR2B, GluR1, and GluR2/3. We observed no significant change in the expression levels of these glutamate receptor subunits after cabergoline treatment for 24 hours. In addition to glutamate receptors (well known as postsynaptic proteins), the levels of synapsinI (a presynaptic protein) were not changed (Fig. 6A). When cell surface expression of these glutamate receptors was determined, significant changes in the levels of NR2B, GluR1, and GluR2/3 were not observed, although NR2A expression was slightly reduced by cabergoline (Fig. 6B).

**Figure 7:**
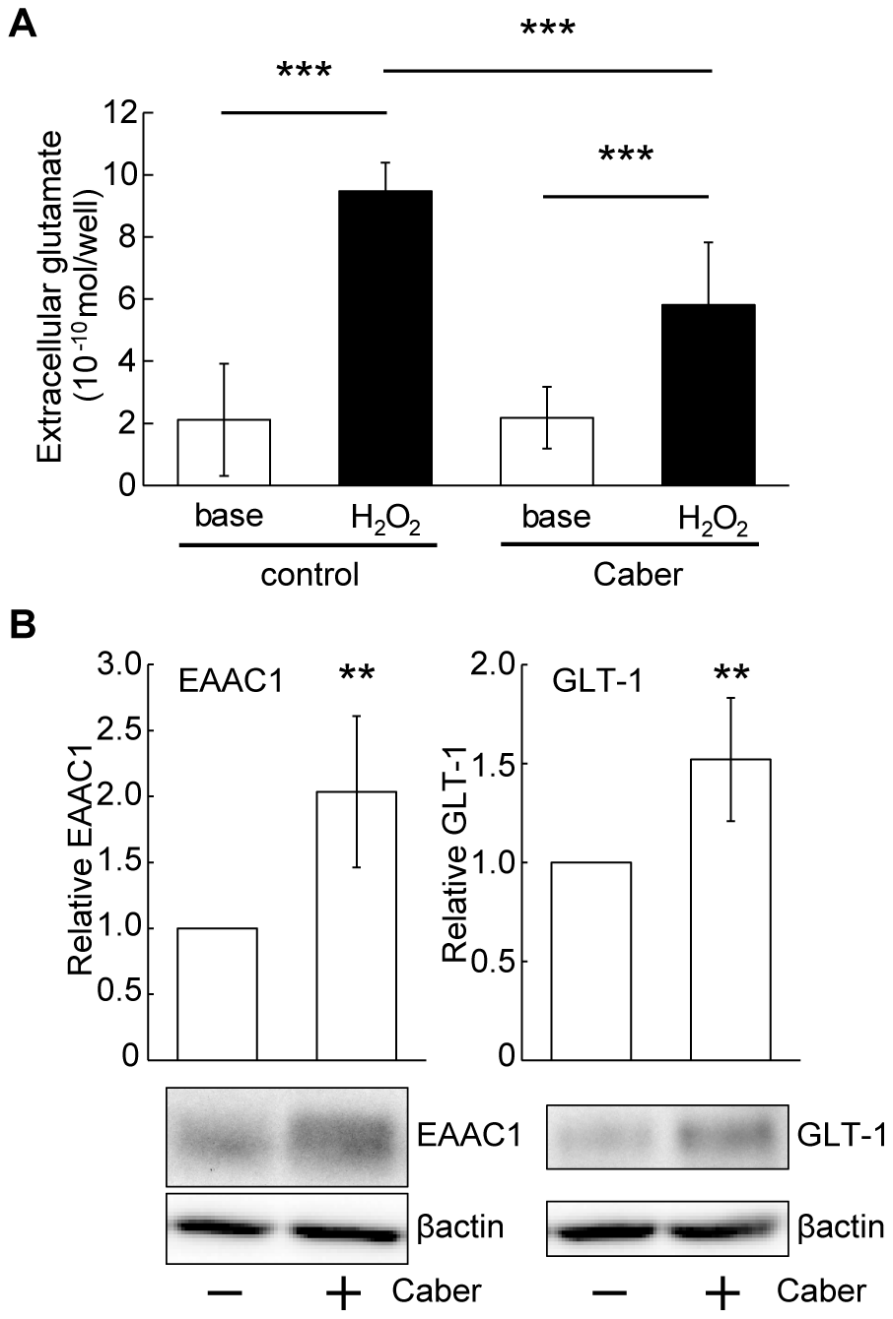
Cabergoline induced upregulation of glutamate transporters. (A) Pretreatment with cabergoline reduced H_2_O_2_-induced extracellular glutamate accumulation. Pretreatment with cabergoline was performed 24 hours before H_2_O_2_ application. After the buffer was collected for measurement of basal concentrations, cells were exposed to buffer containing H_2_O_2_ (50 µM) for 20 min. The data represent mean ± SD (n = 12, n indicates the number of wells per plate for each experimental condition). ***P<0.001 (two-way ANOVA). (B) Cabergoline increased expression levels of glutamate transporters, EAAC1 and GLT-1. Total expression of EAAC1 and GLT-1 after cabergoline treatment for 24 hours. *Uppe*r: Quantification of EAAC1 and GLT-1 expression. All data represent mean ± SD (EAAC1: n = 6, GLT-1: n = 5). **P<0.01 vs. - Caber (*t*-test).

To further determine the protective effect of cabergoline against excitotoxicity, extracellular glutamate concentration was quantified. Interestingly, cabergoline treatment for 24 hours inhibited the increase of glutamate concentration caused by H_2_O_2_ (20 min) (Fig.7A), implying that survival promotion by cabergoline may be attributable, at least in part, to the decreased levels of extracellular glutamate. It is well recognized that glutamate transporters have a critical role in the clearance of glutamate in the synaptic cleft through the uptake of extracellular glutamate. GLAST (glutamate aspartate transporter), GLT-1 (glutamate transporter 1), EAAC1 (excitatory amino acid carrier 1), EAAT4 (excitatory amino acid transporter 4), and EAAT5, have been cloned, and GLT-1 and EAAC1 were shown to have extensive expression throughout the CNS. GLAST and GLT-1 are predominantly expressed in astroglial cells, while EAAC1 is abundantly expressed in neurons [32], [33]. In cultured cortical neurons, high EAAC1 and low GLT-1 expression levels have been reported [33], [34]. Therefore, we measured expression levels of EAAC1 and GLT-1 after cabergoline treatment. Interestingly, cabergoline increased expression levels of both EAAC1 and GLT-1 in cortical cultures (Fig. 7B), implying that the cabergoline-induced upregulation of glutamate transporters has a role in decreasing extracellular glutamate concentration.

## Discussion

In the present study, we found that cabergoline prevented the oxidative stress-induced cell death of cultured cortical neurons via a D_2_ receptor-mediated mechanism. Cabergoline suppressed the activation of ERK signaling, which might have a role in the neuroprotection. Moreover, we found that cabergoline significantly repressed extracellular glutamate accumulation triggered by oxidative stress, and increased the expression of glutamate transporters including EAAC1, which is known to be involved in the clearance of extracellular glutamate.

MAPK cascades, including ERK-, JNK-, and p38-signaling, consist of a family of protein kinases that phosphorylate specific serine and/or threonine sites in target protein substrates in response to changes in extracellular environment, and which regulate various cellular functions including cell survival and death [35], [36]. JNK and p38, called stress-activated protein kinases (SAPK), are strongly activated by stress stimuli (i.e., bacterial lipopolysaccharide, inflammatory cytokines, excitotoxicity mediated by glutamate, and oxidative stress) to induce pro-apoptotic signaling [36], [37]. The ERK pathway has contrary functions including both pro-survival and pro-apoptotic signaling, depending on the type of extracellular stimuli. Neurotrophic factors evoke ERK activation and promote proliferation, differentiation, neurite outgrowth and survival, while stress stimuli induce ERK activation that mediates cell death [38]–[43]. Here, we first examined whether cabergoline increased the expression of BDNF as we previously reported that upregulation of BDNF in hippocampal tissues by cabergoline [21]. Furthermore, BDNF has neuroprotective effects via the activation of Akt and ERK signaling [38]. However, in our present system, cabergoline treatment did not increase the expression of BDNF mRNA (Fig. S6). Therefore, to clarify the mechanism underlying neuroprotection by cabergoline, we examined whether MAPK signaling pathways promoted death signals under H_2_O_2_ exposure. As expected, oxidative stress by H_2_O_2_ significantly stimulated ERK and p38, but not JNK (Fig. 3A), and the activations of both ERK and p38 contributed to neuronal cell death (Fig. 3B). Reports concerning the effect of H_2_O_2_ on JNK in cortical cultures are mixed. One report showed that 100 µM H_2_O_2_ caused a 5-fold increase of pJNK levels [37], while another reported that 1 mM, but not 100 µM, of H_2_O_2_ activated JNK [44]. In our cortical cultures, 50 µM H_2_O_2_ was insufficient to stimulate JNK signaling. This discrepancy between studies might be due to differences in culture conditions (cell density or medium for maintenance). Interestingly, activation of both ERK and p38 was suppressed by pretreatment of cabergoline (Fig. 4A, B). When we performed co-treatment with cabergoline and U0126 to block ERK signaling, there was no additional or synergistic effect compared with the application of U0126 alone. In contrast, co-application of cabergoline and SB203580 (to inhibit p38) exhibited an additive protective effect compared with each chemical alone. Thus, cabergoline might protect cortical neurons predominantly via inhibiting stress-dependent ERK activation although p38 may also be involved in the cell death induction by oxidative stress.

It is well known that ergot-derived D_2_-like receptor agonists (including bromocriptine and pergolide) have a radical scavenging effect [13]. Cabergoline was shown to function as a radical scavenger and that D_2_ receptor antagonists have no effect on neuroprotection by cabergoline [15], [18]. In our system, however, adequate pretreatment time before the addition of H_2_O_2_ (Fig. 1C) and stimulation of the D_2_ receptor (Fig. 2B) were necessary for the neuroprotective effect, suggesting that a D_2_ receptor-mediated mechanism, but not radical scavenging, mainly contributed to the neuroprotection. Importantly, it was shown that D_2_ receptor-dependent synthesis of glutathione partially contributed to a protective effect in mesencephalic neurons and cell lines [14], [16]. The different dependency on receptor-mediated mechanisms may be attributed to differences in the cell-types used, cortical neurons vs mesencephalic neurons/cell line.

Unlike mesencephalic neurons that are composed of monoaminergic neurons and GABAergic neurons, glutamatergic neurons are a major population of the cortical neurons [45]. In glutamatergic neurons, oxidative stress causes excessive extracellular glutamate accumulation that induces excitotoxicity [29], [30]. Such glutamate accumulation and subsequent Ca^2+^ over-load cause the production of ROS because of mitochondria dysfunction and/or the reduction of intracellular antioxidants [43], [46], indicating a close relationship between oxidative stress and excitotoxicity. Notably, the potential involvement of oxidative stress and/or excitotoxicity in the pathogenesis of AD, HD, ALS, and ischemic stroke has been demonstrated [8]–[11]. Moreover, a close relationship between glutamate and ERK signaling was shown. NMDA stimulates ERK signaling via increased intracellular Ca^2+^ through NMDA receptors [47]. Furthermore, considering the Ca^2+^ dependency in the activation of ERK after exposure to H_2_O_2_ [48], it is possible that H_2_O_2_-induced glutamate accumulation might have a role, at least in part, in the activation of ERK signaling. In this study, we confirmed that the oxidative-stress-induced excitotoxicity contributed to neuronal cell death (Fig. 5), and found that pretreatment with cabergoline attenuated the oxidative stress-induced excitotoxicity. Therefore, we examined changes in the levels of glutamate receptor expression by cabergoline, because previous studies suggested that downregulation of glutamate receptors has a preventive merits against excitotoxicity [49], [50]. Although a slight decrease in the cell surface expression of NR2A was observed in our cultures, NR2A total expression and the levels of other glutamate receptor subunits (NR2B, GluR1, and GluR2/3) were unchanged after cabergoline treatment (Fig. 6A, B), implying the downregulation of glutamate receptors is unlikely to be involved. Interestingly, we found that cabergoline treatment decreased the accumulation of extracellular glutamate concentrations under H_2_O_2_ exposure (Fig. 7A), and detected the increased levels of glutamate transporters (EAAC1 and GLT-1) (Fig. 7B). High levels of EAAC1 and low levels of both GLT-1 and GLAST, as well as the critical role of EAAC1 in glutamate uptake in cortical cultures was shown [33], [34]. A previous study reported that knockdown of EAAC1 by icv injection of antisense oligonucleotide caused a 43% decrease in hippocampal glutamate transport and epilepsy in rats [51]. Thus, the upregulation of EAAC1 induced by cabergoline might be important in reducing the levels of extracellular glutamate.

**Figure 8:**
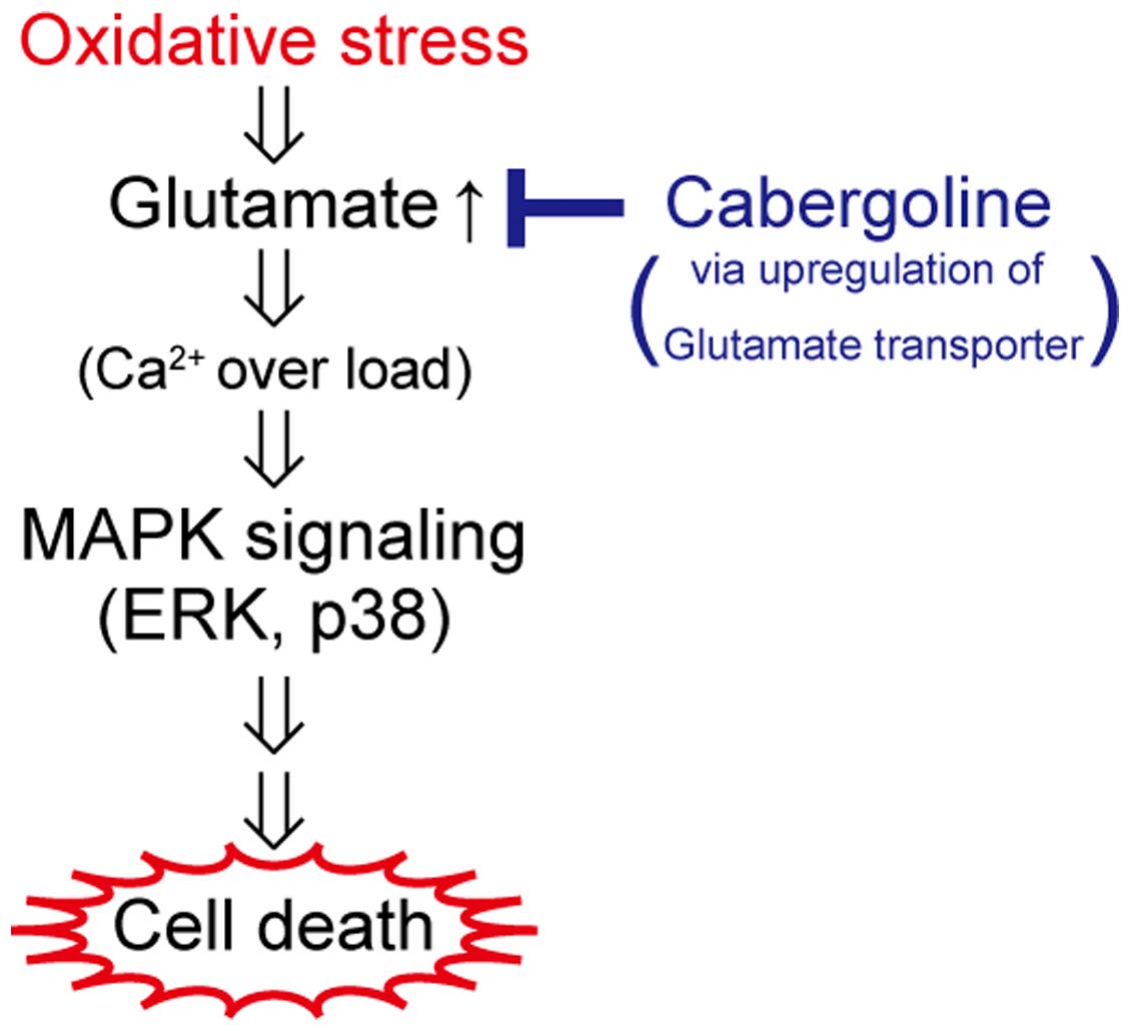
Schematic representation of neuroprotection of cortical neurons by cabergoline. Oxidative stress (H_2_O_2_) causes extracellular glutamate accumulation and subsequent Ca^2+^ over-load in cultured cortical neurons. Then, activated MAPK cascades (ERK and/or p38) are triggered to induce neuronal cell death. Cabergoline treatment decreases extracellular levels of glutamate stimulated by oxidative stress, presumably via upregulation of glutamate transporter (EAAC1/GLT1) expression.

In the present study, we demonstrated cabergoline exerted a neuroprotective effect against oxidative stress in cortical neurons not by scavenging radical species, but rather by the predominant suppression of ERK signaling. To our knowledge, this is the first study to report the upregulation of glutamate transporters and decreased excitotoxicity in cortical neurons by cabergoline. Fig. 8 shows a potential mechanism of the neuroprotective effect of cabergoline. Therefore, cabergoline might be a useful drug for the treatment of brain diseases such as AD, ischemia and PD, where oxidative stress-induced degeneration of cortical neurons is implicated. Furthermore, not only direct scavenging ROS, but also blockade of specific pathways including ERK signaling under oxidative stress might be potential targets for new drug development, although further investigation of the relationship between ERK signaling and glutamate transporters is required.

## Supporting Information

Figure S1 **Application of cabergoline alone does not affect cell viability.** Cortical neurons were exposed to cabergoline at 10 or 50 µM for 36 hours in the absence of H_2_O_2_. MTT assay. The data represent mean ± SD (n = 5–6). Statistics determined by one-way ANOVA.
(TIF)

Figure S2 **Spiperone treatment does not affect cell viability in the presence or absence of H_2_O_2_.** Spiperone (10 µM) was added 24 hours before H_2_O_2_ (50 µM) application. MTT assay was performed. Black bars indicate H_2_O_2_ application. The data represent mean ± SD (n = 6). Statistics determined by two-way ANOVA.
(TIF)

Figure S3 **Influence of U0126 or SB203580 on cell viability in the absence of H_2_O_2_.** No change in cell viability was observed following the addition of U0126 or SB203580. U0126 or SB203580 were added at 10 µM, respectively. After 24 hours, MTT assay was performed to estimate cell viability. The data represent mean ± SD (n = 7). Statistics determined by *t*-test.
(TIF)

Figure S4 **Effect of SP600125, a JNK inhibitor, on cell survival.** SP600125 (5 µM) was added 20 min before H_2_O_2_ (50 µM) application. MTT assay was performed. SP600125 exerted a slight neuroprotection. The data represent mean ± SD (n = 6). ***P<0.001 vs. - H_2_O_2_ - SP600125, ††*P*<0.01 vs. + H_2_O_2_ - SP600125 (two-way ANOVA).
(TIF)

Figure S5 **Cell death induction by glutamate.** Glutamate was added at 10, 50, or 500 µM. After 24 hours, MTT assay was performed. The data represent mean ± SD (n = 8). ***P<0.001 vs. - glutamate (one-way ANOVA).
(TIF)

Figure S6 **Levels of BDNF mRNA after cabergoline treatment in cortical cultures.** Cortical neurons were exposed to cabergoline (10 µM, for 3 or 24 hours). Quantification of BDNF mRNA was carried out with qRT-PCR. KCl (50 mM) stimulation for 3 hours was used as a positive control. The data represent mean ± SD (n = 6). ***P<0.001 vs. con (two-way ANOVA). con∶control.
(TIF)
