# Supplementary figures and images for: Cabergoline, Dopamine D2 Receptor Agonist, Prevents Neuronal Cell Death under Oxidative Stress via Reducing Excitotoxicity
Source: PLoS One. 2014 Jun 10;9(6):e99271. doi: 10.1371/journal.pone.0099271 (PMC4051758; doi:10.1371/journal.pone.0099271)

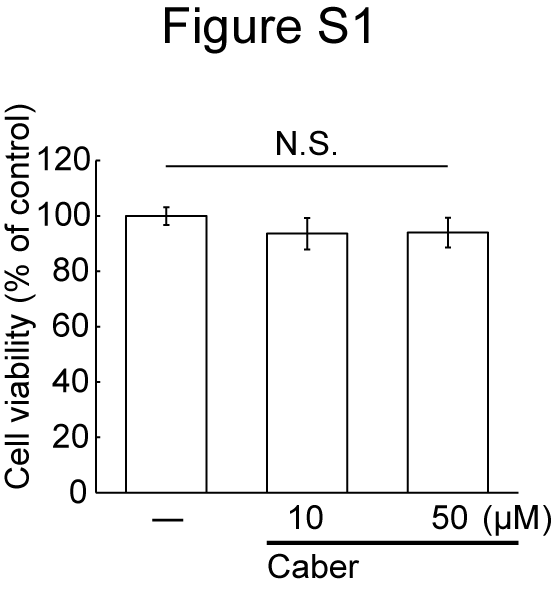

Supplement: Figure S1 — Application of cabergoline alone does not affect cell viability. Cortical neurons were exposed to cabergoline at 10 or 50 µM for 36 hours in the absence of H2O2. MTT assay. The data represent mean ± SD (n = 5–6). Statistics determined by one-way ANOVA. (TIF) [file pone.0099271.s001.tif]

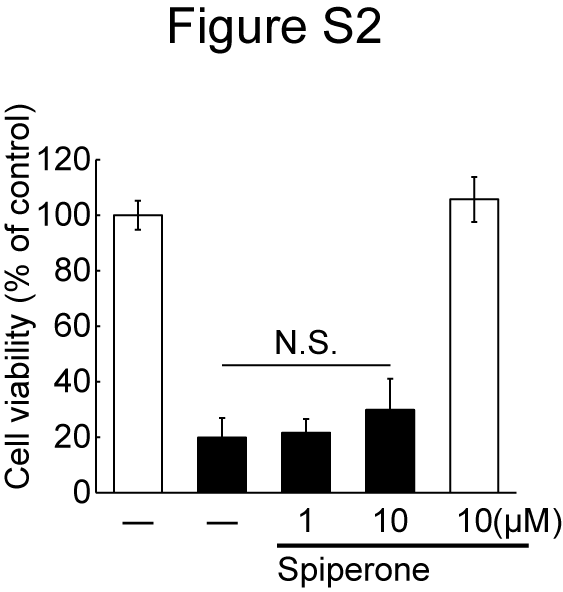

Supplement: Figure S2 — Spiperone treatment does not affect cell viability in the presence or absence of H2O2. Spiperone (10 µM) was added 24 hours before H2O2 (50 µM) application. MTT assay was performed. Black bars indicate H2O2 application. The data represent mean ± SD (n = 6). Statistics determined by two-way ANOVA. (TIF) [file pone.0099271.s002.tif]

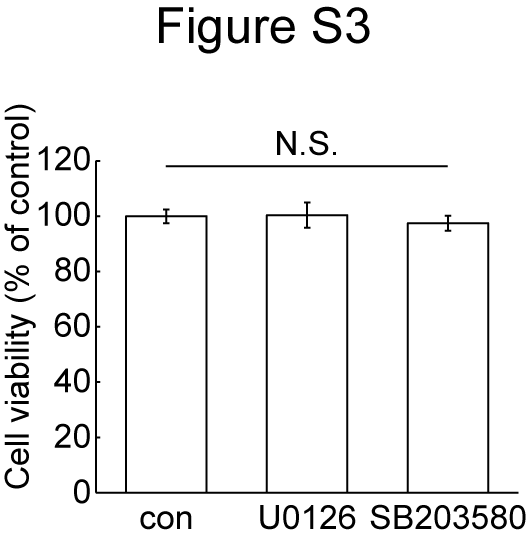

Supplement: Figure S3 — Influence of U0126 or SB203580 on cell viability in the absence of H2O2. No change in cell viability was observed following the addition of U0126 or SB203580. U0126 or SB203580 were added at 10 µM, respectively. After 24 hours, MTT assay was performed to estimate cell viability. The data represent mean ± SD (n = 7). Statistics determined by t-test. (TIF) [file pone.0099271.s003.tif]

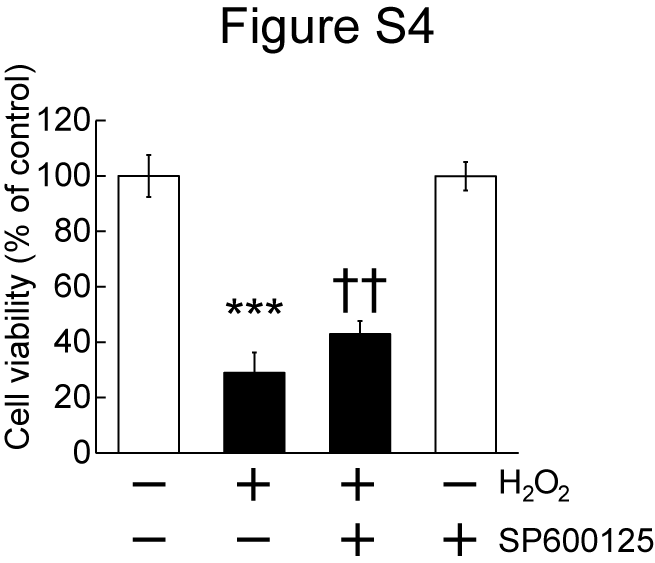

Supplement: Figure S4 — Effect of SP600125, a JNK inhibitor, on cell survival. SP600125 (5 µM) was added 20 min before H2O2 (50 µM) application. MTT assay was performed. SP600125 exerted a slight neuroprotection. The data represent mean ± SD (n = 6). ***P<0.001 vs. - H2O2 - SP600125, ††P<0.01 vs. + H2O2 - SP600125 (two-way ANOVA). (TIF) [file pone.0099271.s004.tif]

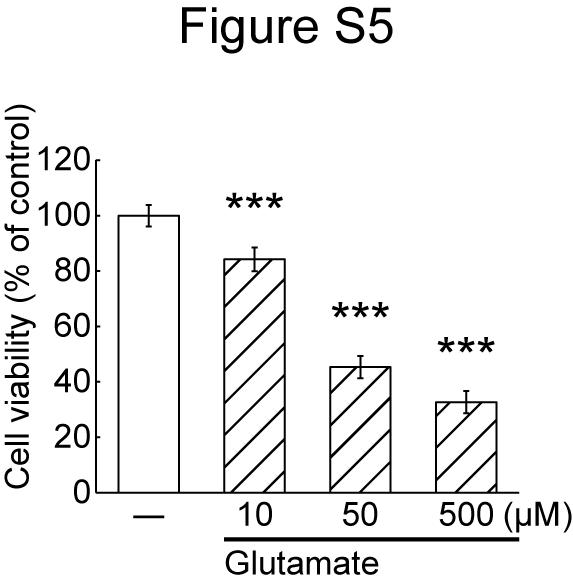

Supplement: Figure S5 — Cell death induction by glutamate. Glutamate was added at 10, 50, or 500 µM. After 24 hours, MTT assay was performed. The data represent mean ± SD (n = 8). ***P<0.001 vs. - glutamate (one-way ANOVA). (TIF) [file pone.0099271.s005.tif]

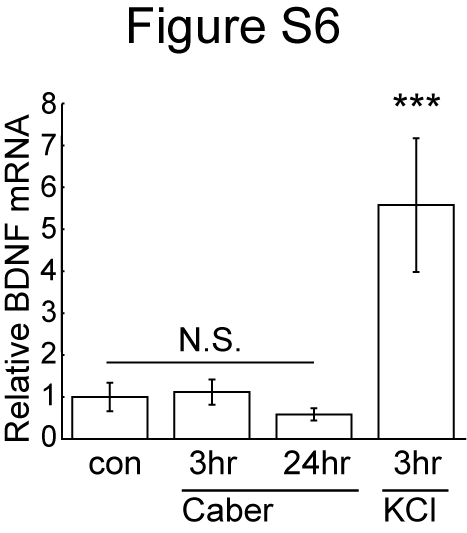

Supplement: Figure S6 — Levels of BDNF mRNA after cabergoline treatment in cortical cultures. Cortical neurons were exposed to cabergoline (10 µM, for 3 or 24 hours). Quantification of BDNF mRNA was carried out with qRT-PCR. KCl (50 mM) stimulation for 3 hours was used as a positive control. The data represent mean ± SD (n = 6). ***P<0.001 vs. con (two-way ANOVA). con∶control. (TIF) [file pone.0099271.s006.tif]
